# The genome sequence and genomic diversity of soybean tentiform leafminer (*Macrosaccus morrisella*)

**DOI:** 10.1093/g3journal/jkaf021

**Published:** 2025-02-04

**Authors:** Christopher Faulk, Arthur V Ribeiro, Carrie Walls, Robert L Koch

**Affiliations:** Department of Animal Science, University of Minnesota, Saint Paul, MN 55108, USA; Department of Entomology, University of Minnesota, Saint Paul, MN 55108, USA; Department of Animal Science, University of Minnesota, Saint Paul, MN 55108, USA; Department of Entomology, University of Minnesota, Saint Paul, MN 55108, USA

**Keywords:** soybean, leafminer, nanopore, genome assembly, population genomics

## Abstract

The soybean tentiform leafminer, *Macrosaccus morrisella* (Fitch) (Lepidoptera: Gracillariidae), is native to North America where it was known to feed on American hogpeanut and slickseed fuzzybean. However, it has recently expanded its host range to include soybean, an important agricultural crop. Here, we report a new, highly contiguous genome for this species with a length of 245 Mb, N50 of 9 Mb, and 96.33% BUSCO completeness. The mitochondrial genome shares only 81% identity to its nearest relative in the NCBI nucleotide database indicating long-standing divergence or sparse sequencing in this clade. To determine whether host plant choice is genetically driven, we sequenced 18 individuals across 3 locations in Minnesota, USA, collected from both American hogpeanut and soybean plants. Genetic variation did not correlate with population structure based on either geography or host plant species (weighted *F*_ST_ estimate: 0.0058). As a secondary measure, we independently assembled complete mitochondrial genomes from all individuals and observed no delineation between host or location. The overall lack of detectable population structure at the nuclear and mitochondrial genome levels suggests a large population with flexible dietary preferences and does not show evidence of genetically driven host preference.

## Introduction

Gracillariidae is a diverse family of about 2000 species whose larvae are plant miners, with most species being monophagous (eating a single host species) or oligophagous (eating only a few species) leaf miners (Davis 1987; van Nieukerken *et al*. 2011; Kawahara *et al*. 2017; De Prins and De Prins 2024) Gracillariid larvae are hypermetamorphic wherein instars of different developmental stages have distinct sap-feeding and tissue-feeding forms (Davis 1987; De Prins and De Prins 2024). Within this family, *Macrosaccus* is a relatively small genus of 5 New World species that feed on Fabaceae (Fabales) (Davis and De Prins 2011). The soybean tentiform leafminer, *Macrosaccus morrisella* (Fitch) (Lepidoptera: Gracillariidae), is native to North America where it was known to feed on American hogpeanut, *Amphicarpaea bracteata* (L.) Fernald, and slickseed fuzzybean, *Strophostyles leiosperma* (Torr. and A. Gray) Piper (Fabales: Fabaceae) (Davis and De Prins 2011). These 2 vining legumes occur in forest understories in eastern North America (USDA 2024a). However, *M. morrisella* was recently discovered feeding on soybean in Quebec, Canada, in 2016 and in Minnesota, USA, in 2021 (Koch *et al*. 2021). Soybean is a major source of vegetable oil and protein grown on 34,913,000 and 2,300,000 ha in the USA and Canada, respectively (USDA 2024b).

Adults of *M. morrisella* oviposit on soybean leaves (Menger *et al*. 2024). The feeding injury (i.e. mines) created by larvae of *M. morrisella* progress from serpentine mines, to blotch mines to tentiform mines as the larvae develop (Davis and De Prins 2011; Koch *et al*. 2021; Menger *et al*. 2024). These mine types are named after the appearance of the damage on the leaf as the miner feeds and the ultimate tentiform structure is created. The larvae pupate inside the mines (Davis and De Prins 2011; Koch *et al*. 2021). Like other leafminers (Kappel and Proctor 1986; Liu *et al*. 2015; Ullah *et al*. 2020), it is believed that the feeding injury from *M. morrisella* reduces photosynthesis of infested leaves of soybean plants (Koch *et al*. 2021). Development of *M. morrisella* from egg to adult requires an accumulation of 425 degree-days above a lower developmental threshold of 8.96°C, which could enable repeated attack of soybean plants by multiple generations of *M. morrisella* within a single growing season (Ribeiro *et al*. 2024).

As a new pest, relatively little is known about the biology of *M. morrisella*. Kogan (1981) stated that an important source of new pests for soybean in North America would be native oligophagous (i.e. legume feeding) insects adapting to soybean, which seems to align well with the appearance of *M. morrisella* in soybean. Advances in genomics research are facilitating the understanding of insect adaptation to new host plants (Simon *et al*. 2015). However, despite their biological and economic significance, the genomes have been sequenced from relatively few (only 3) species of Gracillariidae. Here, we provide the first genome sequence for *M. morrisella* to establish a foundation for examination of its biology and development of more sustainable management programs. In addition, we characterize the genomic variability and association with host plant use.

## Materials and methods

### Sample collection

To sequence the complete genome of *M. morrisella*, individuals were obtained from a laboratory colony established in 2022 and maintained over ∼22 generations according to Menger *et al*. (2024) at the University of Minnesota, Minnesota, USA. In short, this colony was initiated with adults of *M. morrisella* reared from soybean leaves infested with pupae of *M. morrisella* collected from a soybean field in Henderson, Minnesota, USA, in summer 2022. In the laboratory colony, emerged adults were allowed to oviposit for 48–72 h on potted soybean plants (variety: “Sheyenne”) at the V1–V2 developmental stage (Purcell *et al*. 2014) inside an oviposition cage. After oviposition, potted plants with *M. morrisella* eggs were transferred to a separate cage for development of immature *M. morrisella*. When *M. morrisella* reached the pupal stage, the pots were transferred to an adult emergence cage. After adult emergence, the process described above was repeated to maintain the colony. The colony was maintained in a walk-in environmental growth chamber at 25 ± 2°C and 16 h photophase.

From this colony, pupae were manually collected from mines of infested soybean plants with the help of an entomological pin (size #1) and a fine paintbrush to carefully open the mines and the silken cocoons. Collected pupae were placed inside individual 2-mL microcentrifuge tubes for adult emergence. A single pair of freshly emerged (i.e. <24 h old) adults (F0) was bred using the above-mentioned methods, their offspring were raised until pupation, and an F1 sibling pair was bred using these same methods. Finally, the resulting F2 offspring were used for sequencing the reference genome and are referred to as the “F2 isoline.”

To examine population genomics of *M. morrisella*, soybean fields infested with *M. morrisella* were sampled on July 20 and 2023 August 10 in Brooten, Minnesota, on 2023 June 22 and 23 in St. Paul, Minnesota, and on 2023 August 14 in Rochester, Minnesota, USA. For each field on each sample date, 20 soybean leaflets containing tentiform mines of *M. morrisella* were manually collected from field edges (first 3 rows) adjacent to wooded areas, placed inside an individually labeled 17 × 17-cm resealable plastic bag, and then into a refrigerated cooler for transportation to the laboratory at the University of Minnesota Saint Paul Campus. Samples were collected from edges of soybean fields near wooded areas because infestations are higher at these locations (Ribeiro and Koch 2024). Similarly, 20 leaflets of American hogpeanut containing tentiform mines of *M. morrisella* were collected from forested areas <2.5 km from the soybean fields sampled, on the same dates and using the same methods described above for soybean. These methods were used to minimize geographical and temporal variability between samples from both hosts. In the laboratory, the collected leaflets were placed inside emergence cages similar to Melotto *et al*. (2023), with a moist cotton ball on a petri dish inside each cage to help maintain the relative humidity inside cages at ∼70%. Deionized water was added to the cotton balls every 2–3 days. Cages were maintained inside a rearing room at ∼25°C and 16 h photophase for adult emergence. The emergence of *M. morrisella* adults was evaluated every 2–3 days, and adults were collected from emergence cages with the help of a mechanical aspirator (Clarke Mosquito Control, #13500, St. Charles, IL, USA). On each evaluation day, collected adults were placed inside individual 2-mL microcentrifuge tubes containing 90% ethanol/deionized water (v/v) solution and stored inside a −20°C freezer. The preserved adults of *M. morrisella* were used for population genomics analysis described below.

### DNA extraction and sequencing

Approximately 20 individuals from the F2 isoline underwent DNA extraction using a Qiagen MagAttract kit (cat. 67563) according to the manufacturer's instructions. The resulting DNA was pooled and library prepped using a ligation sequencing kit, LSK-114 from Oxford Nanopore Technologies (ONT). The library was run on a single promethION flowcell using a P2 Solo instrument (ONT). Resulting squiggle data in pod5 format were basecalled using dorado v0.7.0 and model dna_r10.4.1_e8.2_400bps_sup@v4.3.0 including 5mC methylation calling at CpG sites.

### Genome assembly

Reads longer than 5 kb were corrected using the HERRO algorithm as implemented in dorado v0.7.0. The genome was assembled using hifiasm v0.16.0 (Cheng *et al*. 2021). Completeness was assessed using Compleasm (Huang and Li 2023), an alternative to BUSCO that uses the same BUSCO databases. Here, we used the lepidoptera_odb10 database matching to 5286 genes for completeness. To reduce duplicate BUSCO percentage below 1%, we applied three successive rounds of purge_dups v1.2.6 (Guan *et al*. 2020). Residual adaptor and vector sequence was removed from the assembly using the NIH Foreign Contaminant Screening tool. Repeats were identified using RepeatModeler2 v2.0.5 (Flynn *et al*. 2020) to generate a species-specific repeat database which was run against the assembly using RepeatMasker v4.1.6 (Smit *et al*. 2013). Other genomes from Gracillariidae underwent the same analysis for comparative purposes. The mitochondrial sequences were identified using MitoHiFi (Uliano-Silva *et al*. 2023) and initially aligned to the mitogenome from *Euspilapteryx auroguttella* Stephens (Lepidoptera: Gracillariidae). DNA methylation was determined by pileup of the modified cytosines into bedmethyl format via modkit v0.3.1. Protein prediction and annotation was performed using GeMoMa v1.9 with monarch butterfly genes as a guide. The pipeline for assembly and analysis is available in Supplementary File 1.

### Population genomic analysis

The DNA of the field populations was extracted and sequenced with the same parameters as above. Each individual was barcoded individually and sequenced using the native barcoding kit SQK-NBD-114 from ONT. Resulting barcodes were separately aligned to the reference isoline genome. SNVs were called using Clair3 v1.0.10 (Zheng *et al*. 2022) and resulting vcf files were merged using bcftools (Danecek *et al*. 2021). The merged vcf was filtered to remove any variants below Q20, indels, no more than 10% individuals missing a genotype, minimum depth 2X, max depth 100X, and a minor allele frequency of <8% (at least 3 individuals must have a minor allele present per variant location). *F*_st_, principal component analysis, heterozygosity, and depth were performed in plink 1.9 (Purcell *et al*. 2007). First linkages were pruned and the filtered vcf was used to create the principal component analysis (PCA) plot. Admixture plots were created using admixturePipeline (Mussmann *et al*. 2023) and visualized with Clumpak (Kopelman *et al*. 2015). The VCF file for all individuals is available as Supplementary File 2.

## Results and discussion

### Assembly

Pairs of *M. morrisella* from a laboratory colony were selected for sibling mating to reduce heterozygosity prior to reference genome assembly. After 2 generations, larval specimens were collected for DNA extraction, sequencing, and assembly. Nanopore sequencing produced 79.6 Gb of reads with a read length N50 of 3.7 kb and an average *Q*-score of 16.9 (Supplementary Table 1). The complete output underwent read correction and filtering resulting in 21 Gb with a read N50 of 9.1 kb used for assembly. The assembled F2 isoline reference genome (STLf2iso) is 245 Mb in length, consisting of 68 contigs with an N50 of 9 Mb and is similar or better in quality to members of the same insect family (Table 1). The genome contained 96.33% BUSCOs (lepidoptera_odb10) with <1% duplication rate, similar to 2 of the other high-quality Gracillariidae genomes available on NCBI and exceeding the cocoa pod borer, *Conopomorpha cramerella* (Snellen) (Lepidoptera: Gracillariidae) assembly (Table 2). The initial genome assembly was attempted using wild-caught specimens; however, difficulty in achieving high contiguity led us to develop the F2 isoline to reduce heterozygosity. Our assembly pipeline deviated from standard practice due to the high heterozygosity causing assemblers to generate excessive haplotigs, manifested by extraordinarily high duplicate BUSCOs in draft assemblies (some assemblies with 95% duplicate rates) and abnormally large estimated genome sizes. The use of HERRO-corrected reads to improve read quality, the choice of hifiasm over flye assembler, and 3 rounds of haplotig purging via purge_dups resulted in a highly contiguous genome with <1% duplicate BUSCOs, and the expected genome size for a leafminer moth of this family.

**Table 1.** Assembly comparison of *M. morrisella* and other gracillariid genomes available from NCBI.

Scientific name	Common name	Assembly	Num seqs	Sum length	Min length	Max length	N50	N50 num	GC (%)
*Macrosaccus morrisella*	Soybean tentiform leafminer	STLf2iso	68	245,695,242	475	16,440,956	9,036,253	11	36.44
*Conopomorpha cramerella*	Coco pod borer	ASM1293212v1	73,142	497,288,140	200	351,205	12,136	7,764	38.13
*Euspilapteryx auroguttella*	Leaf blotch miner	ilEusAuro2.1	103	331,914,766	9,628	22,118,606	11,650,979	12	37.39
*Aspilapteryx tringipennella*	Leaf blotch miner	ilAspTrin1.1	144	261,726,888	1,000	17,401,394	9,501,776	12	37.44

**Table 2.** BUSCO completeness of *M. morrisella* and other gracillariid genomes available from NCBI.

Scientific name	Genome	Complete	Single	Duplicate	Fragment (1)	Fragment (2)	Missing
*Macrosaccus morrisella*	STLf2iso	96.33%	95.76%	0.57%	0.25%	0.00%	3.42%
*Conopomorpha cramerella*	ASM1293212v1	75.22%	66.78%	8.44%	11.39%	0.25%	13.15%
*Euspilapteryx auroguttella*	ilEusAuro2.1	97.47%	96.94%	0.53%	0.36%	0.00%	2.18%
*Aspilapteryx tringipennella*	ilAspTrin1.1	98.07%	97.67%	0.40%	0.28%	0.00%	1.65%

The genome consists of 42.28% repetitive elements with the vast majority being interspersed repeats rather than simple repeats (Table 3). Most were novel to this genome with only 4.35% detected when using the publicly available Dfam database. The repeat content is in line with other animal genomes, though the elements which make up the bulk of the retroposed copies appear to be novel to this clade and were not represented in the public database of known repeat elements.

**Table 3.** Repeat content of *M. morrisella* genome.

Name	Number of Elements	Combined length (bp)	Genome (%)
*Retroelements*	65,049	15,218,873	6.19
L2/CR1/Rex	11,341	3,125,896	1.27
R1/LOA/Jockey	267	266,376	0.11
R2/R4/NeSL	109	102,121	0.04
RTE/Bov-B	40,620	6,683,247	2.72
*LTR elements*	4,060	3,315,565	1.35
BEL/Pao	1,809	1,259,292	0.51
Ty1/Copia	238	240,322	0.1
Gypsy/DIRS1	2,013	1,815,951	0.74
*DNA transposons*	11,208	2,342,485	0.95
hobo-Activator	492	131,477	0.05
Tc1-IS630-Pogo	1,098	482,457	0.2
PiggyBac	85	89,644	0.04
*Other*	4,572	508,478	0.21
*Rolling-circles*	843	67,541	0.03
*Unclassified*	538,871	86,328,898	35.14
**Total interspersed repeats**	-	**103,890,256**	**42**.**28**
Satellites	6	3,542	0
Simple repeats	58,446	2,606,605	1.06
Low complexity	9,588	468,330	0.19

**Fig. 1.**
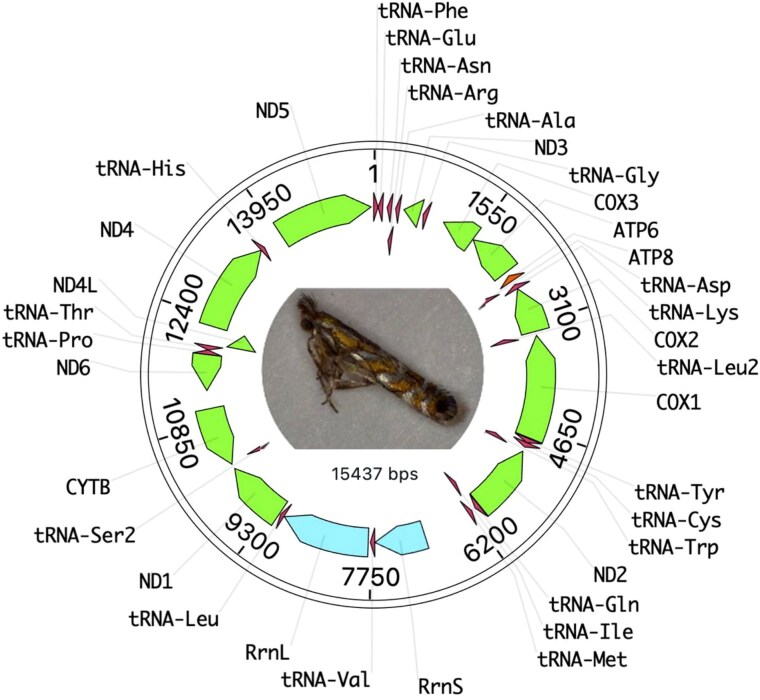
Mitogenome structure of the F2 isoline strain of *M. morrisella*. Coding regions are in green, tRNAs in red, and the small and large mitochondrial ribosomal subunits are in blue.

The mitochondrial genome is 15,437 bp in length with 37 genes with no frameshifts in our assembly (Fig. 1). This is smaller than the bait mitogenome we used for assembly which was 17,050 bp in length from *E. auroguttella.* When BLASTed against lepidoptera, the closest match is to *Argyresthia albistria* Haworth (Lepidoptera: Yponomeutidae) mitochondrion with 81.55% identity over 99% length. When BLASTed against Gracillariidae, the closest match is to *E. auroguttella* with 82.83% identity over 97% length. High mitochondrial divergence even to other Gracillariidae genomes indicates a long history of separation and sparse coverage of sequenced genomes in this clade.

Protein prediction yielded 10,709 transcripts with an overall protein BUSCO score of 90.77% completeness consisting of 84.26% single copy, 6.51% duplicates, 0.19% fragments, 9.04% missing (Supplementary File 3) (Melotto *et al*. 2023). To determine pipeline efficacy, we used the same parameters on the previously published *E. auroguttella* genome (ilEusAuro2.1) and found very similar detection levels, 90.11% completeness consisting of 84.43% single copy, 5.68% duplicates, 0.40% fragments, and 9.50% missing.

### Population genomics

We sequenced 18 individuals collected from the field, representing 3 biological replicates per host plant (either soybean or American hogpeanut), from 3 geographical locations, St. Paul, Rochester, and Brooten, Minnesota. Their depth when mapped to our 245 Mb genome ranged from 6X to 45X, mean 19X (Supplementary Table 2). Nanopore sequencing allows detection of DNA modifications along with sequence variants. None of the 18 individuals had any appreciable DNA methylation (mean 1.01%) or DNA hydroxymethylation (mean 0.22%), indicating that this species does not utilize DNA methylation as a regulatory mechanism (Supplementary Table 3).

We called variants using Clair3 followed by standard filtering guidelines. Variant depth ranged from 4.62X to 24.95X. The fixation index (*F*_ST_) estimate was 0.0052 (weighted *F*_ST_ estimate: 0.0058) indicating very low genetic differentiation between groups. The populations are largely similar genetically and are likely interbreeding with high gene flow or share a very recent common ancestor, leading to similar genetic structures (Table 4).

**Table 4.** Population genomics of *M. morrisella* from soybean or American hogpeanut collected at 3 geographical locations in Minnesota, USA.

ID	Clair3 variants	Mean depth	Observed (homozygous)	Estimated (homozygous)	Filtered sites	*F^a^*
St.Paul_Hogpeanut_1	7,683,179	5.70	96,055	101,804.3	138,751	−0.15561
St.Paul_Hogpeanut_2	9,128,991	13.70	107,412	109,066.9	148,306	−0.04218
St.Paul_Hogpeanut_3	8,989,974	8.27	104,614	108,517.2	147,600	−0.09987
St.Paul_Soybean_1	9,081,916	12.08	107,633	109,077.2	148,328	−0.03679
St.Paul_Soybean_2	9,215,482	13.09	107,833	108,825.9	147,972	−0.02536
St.Paul_Soybean_3	9,331,466	16.66	108,591	108,923.1	148,099	−0.00848
Rochester_Hogpeanut_1	9,145,820	10.09	106,582	108,638.5	147,735	−0.0526
Rochester_Hogpeanut_2	9,369,275	19.50	107,009	108,828.1	147,987	−0.04645
Rochester_Hogpeanut_3	9,202,282	11.01	107,356	108,773.8	147,904	−0.03623
Rochester_Soybean_1	9,393,790	24.65	107,055	108,757.2	147,873	−0.04352
Rochester_Soybean_2	9,515,691	24.95	107,623	108,876	148,017	−0.03201
Rochester_Soybean_3	8,267,404	7.08	103,034	106,543.9	144,939	−0.09141
Brooten_Hogpeanut_1	7,772,523	6.07	98,226	106,014.2	144,339	−0.20322
Brooten_Hogpeanut_2	6,768,449	4.77	88,920	96,074.6	131,140	−0.20404
Brooten_Hogpeanut_3	6,572,378	4.62	86,391	97,063.6	132,512	−0.30107
Brooten_Soybean_1	8,234,980	7.22	101,119	108,277	147,304	−0.18341
Brooten_Soybean_2	9,164,946	20.86	108,515	108,789.3	147,920	−0.00701
Brooten_Soybean_3	9,103,057	12.69	108,255	108,728.5	147,829	−0.01211
Isoline	3,797,437	43.00	-	-	-	-
Mean	8,407,318	14.00	103,457	106,754	145,253	−0.0878539

^a^Negative values indicate excess of heterozygotes beyond HW eq.

The heterozygosity was calculated on filtered biallelic SNVs of high quality. The moderate negative inbreeding coefficient (*F* values) in the field samples suggests that they are generally outbred, with a small excess of heterozygous sites, which is typical for natural populations. Most field samples have *F* values between −0.1 and −0.03, indicating a modest excess of heterozygous sites, which could be due to the Wahlund effect (Garnier-Géré and Chikhi 2013). The Wahlund effect occurs when a population is structured into subpopulations with limited gene flow between them, and these subpopulations have different allele frequencies. When samples are pooled together without accounting for the subpopulation structure, it can result in an apparent excess of heterozygosity across the combined population compared with Hardy–Weinberg equilibrium.

**Fig. 2.**
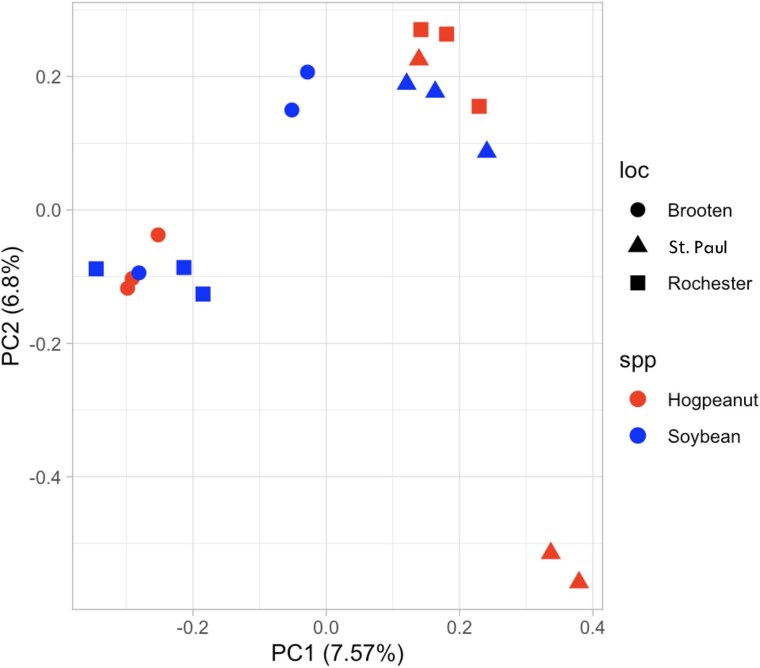
PCA of genetic variation among *M. morrisella* populations. The plot shows 18 individuals grouped by sampling location (Brooten, St. Paul, Rochester) and host plant (American hogpeanut or soybean). PC1 and PC2 explain 7.57 and 6.8% of the variation, respectively.

The PCA plot resulted in 2 distinct clusters, though not separated by either host species or geographic region of collection, indicating wide diversity and unrestricted gene flow in this population (Fig. 2). The variation explained by the first 2 components is relatively low (7.57% for PC1 and 6.8% for PC2), suggesting that there may be other genetic factors influencing the populations that are not captured by these 2 components alone.

**Fig. 3.**
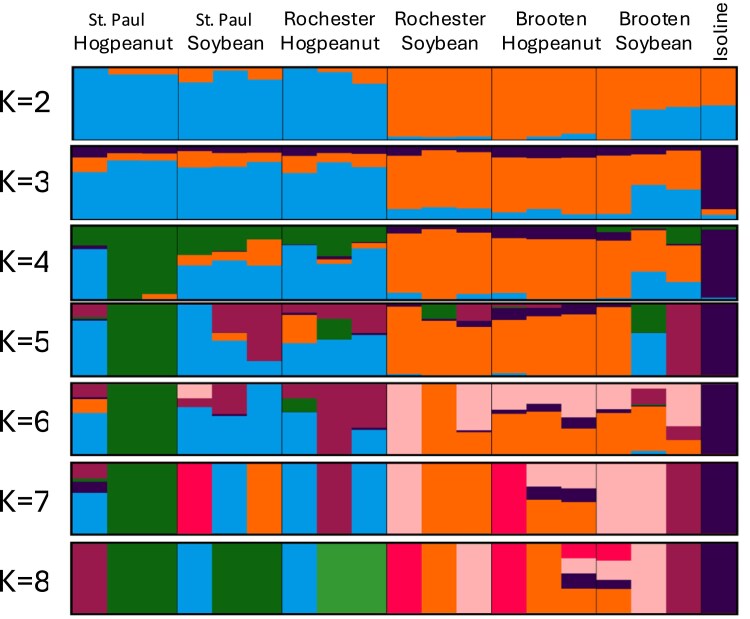
Admixture plot showing genetic structure of *M. morrisella* populations across different locations (St. Paul, Rochester, Brooten) and host plants (American hogpeanut, soybean). Each vertical bar represents an individual, and different colors indicate proportions of ancestry from inferred genetic clusters. The number of clusters (*K*) ranges from 2 to 8, revealing increasing genetic complexity. Clear differentiation based on host plant is visible at lower *K* values, while location-specific variation and admixture become more apparent at higher *K* values.

We performed an admixture analysis to further distinguish the population structure (Fig. 3). At *k* = 2, there was a clear division of populations between the St. Paul and Brooten samples; however, the Rochester samples clustered on both sides of the distribution by host plant. At *k* = 3, the isoline reference genome diverged from all other samples. Up to *k* = 4, the American hogpeanut samples from Brooten and soybean samples from Rochester maintain the greatest homogeneity, with all individuals in the orange group, suggesting a common origin. At *k* = 4 and above, the population structure no longer diverged in a defined way according to sample origin. Even the most homogenous cluster at *k* = 4 is derived from both host plants indicating that genetic divergence is not driven by host preference.

### Mitochondrial population structure

**Fig. 4.**
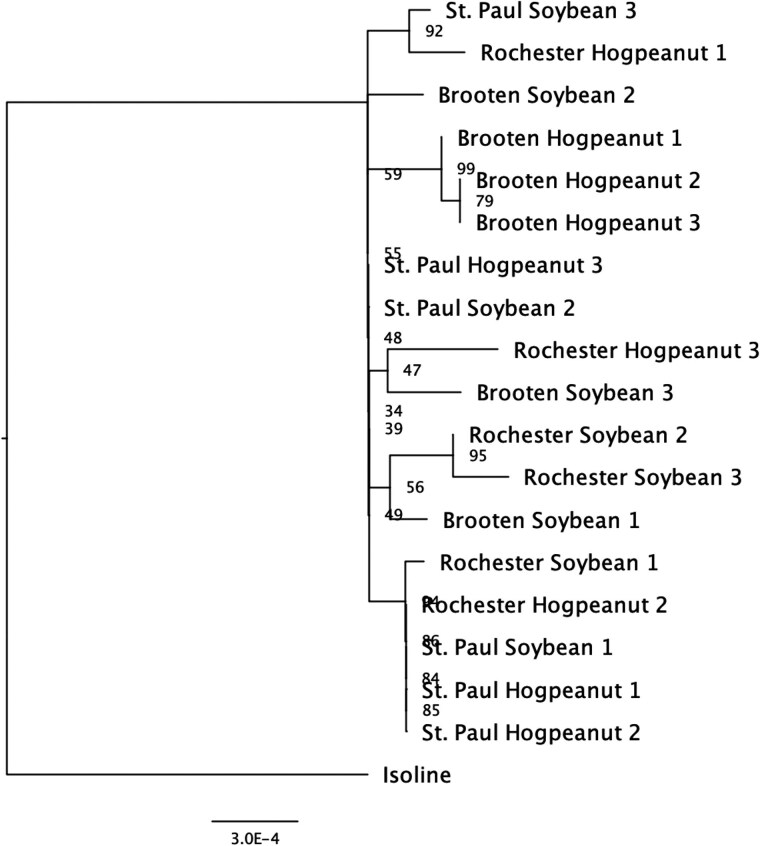
Phylogenetic tree of 18 *M. morrisella* individuals based on mitochondrial genome sequences. Individuals are grouped by sampling location (St. Paul, Rochester, Brooten) and host plant (American hogpeanut, soybean). The tree is rooted with the reference isoline as an outgroup. Some clustering by location is observed (e.g. Brooten and Rochester), but there is no clear pattern by host plant. Bootstrap values indicate support for major branches, with values over 90 suggesting well-supported relationships. Scale bar represents genetic distance.

Genetic divergence between laboratory-reared and wild populations is commonly seen for insects due to factors such as adaptation to laboratory conditions, inbreeding, or genetic drift (Hoffmann and Ross 2018). Mitochondrial sequence divergence was used as a secondary method to reconstruct any population structure that may have existed for *M. morrisella*. We used complete mitogenomes independently assembled from each of the 3 biological replicates collected across host plants and geographic regions (Fig. 4). All field samples clustered separately from the F2 isoline reference mitogenome; however, there was very little sequence divergence among them. No consistent monophyly was seen between host plant and geographic region. The short *x*-axis reflects the small number of substitutions seen over the entire population.

### Conclusion

The newly created reference genome for *M. morrisella* gives insight to its place in the Gracillariidae family tree, showing high genomic heterozygosity and early divergence from related species. The lack of detectable population structure at the nuclear and mitochondrial genome levels suggests a large population with flexible dietary preferences and does not show evidence of genetically driven host preference. Overall, the findings of this study advance the knowledge of the biology of *M. morrisella* and will help to establish the foundation for development of management strategies for this insect.

## Supplementary Material

jkaf021_Supplementary_Data

## Data Availability

Assembly and BioSample information is available at NCBI BioProject number PRJNA1173748, BioSample number SAMN44319150, and accession number JBIOAS000000000. Raw sequencing reads are available at the NCBI sequence read archive accession number SRR32170798. Supplemental material available at G3 online.
