# Supplementary material for: The genome sequence and genomic diversity of soybean tentiform leafminer (Macrosaccus morrisella)
Source: G3 (Bethesda). 2025 Feb 4;15(4):jkaf021. doi: 10.1093/g3journal/jkaf021 (PMC12005143; doi:10.1093/g3journal/jkaf021)
Supplement: jkaf021_Supplementary_Data [file jkaf021_supplementary_data.zip › File_S1_G3-2024-405554.html]

Soybean Tentiform Leafminer


# Soybean Tentiform Leafminer

#### chris faulk

#### 2023-11-11

# Purpose

# Samples

# F2 Isoline Assembly Pipeline

```
# Single promethION R10.4.1 flowcell
# Basecalled by minknow with dorado basecall_model_version_id=dna_r10.4.1_e8.2_400bps_sup@v4.3.0
# Raw data: STL_F2_isoline_pooled_5-13-24
# Passed: 71.45 Gb + 8.2 Gb
# Failed: 11.16 Gb + 1.48 Gb

# Filter for reads > 5 kb
seqkit seq -m 5000 STL_F2_isoline_pooled.fq.gz > STL_F2_isoline_pooled.5k.fq.gz

# Read correction performed with dorado
~/Desktop/dorado-0.7.0-linux-x64/bin/dorado correct STL_F2_isoline_pooled.5k.fq > STL_F2_isoline_pooled.5k.herro.fa

# Assembly with hifiasm
./hifiasm/hifiasm -o STL_F2_isoline_pooled.5k.herro.asm -t 32 STL_F2_isoline_pooled.5k.herro.fa
```

## Purge\_dups

Purge\_dups was run to remove haplotigs and contig overlaps in a de
novo assembly based on read depth.

```
# Align the data to generate paf files
minimap2 -x map-ont consensus.fasta $i | gzip -c - > $i.paf.gz
minimap2-fast -I 4G -x map-ont -t 32 ../purged.fa ~/Desktop/stl_processing/LeafMiner4k.fastq | pigz > stl.paf.gz

# Produce stats and cutoffs file
../purge_dups/bin/pbcstat stl.paf.gz
../purge_dups/bin/calcuts PB.stat > cutoffs 2>calcults.log

# Split consensus and self-align
../purge_dups/bin/split_fa ../assembly.fasta > assembly.split.fa
minimap2 -xasm5 -DP assembly.split.fa assembly.split.fa -t 32 | gzip -c - > assembly.split.self.paf.gz

# Purge dups and haplotigs
../purge_dups/bin/purge_dups -2 -T cutoffs -c PB.base.cov assembly.split.self.paf.gz > dups.bed 2> purge_dups.log

# Get purged primary and haplotigs
../purge_dups/bin/get_seqs -e dups.bed ../assembly.fasta

# Generate histogram
../purge_dups/scripts/hist_plot.py -c cutoffs PB.stat PB.base.png
```

## Add in Mitochondrial genome

```
cat ../MitoHiFi/STL_hifiasm-herro-p3-mitogenome/final_mitogenome.fasta >> purged.fa
mv purged.fa purged-mitogenome.fa
```

## Foreign Contaminant Screen

```
# Required prior to NIH ubmission.
# FCS-adapt removes adapter and vector sequences

# Install
curl -LO https://github.com/ncbi/fcs/raw/main/dist/run_fcsadaptor.sh
chmod 755 run_fcsadaptor.sh
curl https://ftp.ncbi.nlm.nih.gov/genomes/TOOLS/FCS/releases/latest/fcs-adaptor.sif -Lo fcs-adaptor.sif

# Run
sudo ./run_fcsadaptor.sh --fasta-input purged-mitogenome.fa.gz --output-dir ./FCS-output/ --euk --container-engine singularity --image fcs-adaptor.sif

# Clean the genome
curl -LO https://github.com/ncbi/fcs/raw/main/dist/fcs.py
zcat STL_hifiasm-herro-p3-final_mtDNA.fa.gz | sudo python3 ./fcs.py clean genome --action-report ./FCS-output/fcs_adaptor_report.txt --output clean.fasta --contam-fasta-out contam.fasta
```

## Scaffolding

```
# Install ntLink for scaffolding with gap-filling
mamba install -c bioconda -c conda-forge ntlink

# Run with 3 rounds
ntLink_rounds run_rounds_gaps target=clean.fasta reads=STL_F2_isoline_pooled.10k.fa.gz k=32 w=100 t=5 rounds=3
```

## Final Assembly

```
cp FCS-output/ntlink-purged/clean.fasta.k32.w100.z1000.ntLink.3rounds.fa STL-final.fa
```

## QC and BUSCO

```
# Read stats
# Assembly-stats
https://github.com/sanger-pathogens/assembly-stats

# Compleasm
mamba create -n compleasm -c conda-forge -c bioconda compleasm
mamba activate compleasm
compleasm run -t 32 -l lepidoptera -L ~/Desktop/genomes/mb_downloads -a STL-final.fa -o compleasm-STL-final
```

## Repeats

### RepeatModeler

```
# Install Singularity
wget https://github.com/sylabs/singularity/releases/download/v4.1.4/singularity-ce_4.1.4-jammy_amd64.deb

# Install DFAM TEtools
curl -sSLO https://github.com/Dfam-consortium/TETools/raw/master/dfam-tetools.sh
chmod +x dfam-tetools.sh
./dfam-tetools.sh

# Build database
BuildDatabase -name stl-final STL-final.fa

# Run RepeatModeler
RepeatModeler -database stl-final -LTRStruct -threads 32
```

### RepeatMasker

```
# Default library from DFAM
RepeatMasker -spec lepidoptera -s -pa 32 -xsmall -gff -e rmblast STL-final.fa

# Custom library created with RepeatModeler2
RepeatMasker -lib stl-final-families.fa -s -pa 32 -xsmall -gff -e rmblast STL-final.fa
```

## GeMoMa Gene Annotation

Wiki

```
# Install conda environment
mamba create -n gemoma
mamba activate gemoma
mamba install -c bioconda gemoma  
 
# Download GeMoMa anyway b/c we need to set java's memory requirements manually
http://www.jstacs.de/download.php?which=GeMoMa

# Run vs Monarch butterfly annotations. 
java -Xmx50g -jar GeMoMa/GeMoMa-1.9.jar CLI GeMoMaPipeline threads=32 outdir=annotation GeMoMa.Score=ReAlign AnnotationFinalizer.r=NO o=true t=../STL-final.fa.gz i=D_plexippus a=GCF_018135715.1_MEX_DaPlex_genomic.gff.gz g=GCF_018135715.1_MEX_DaPlex_genomic.fna.gz

# Busco on protein mode:
compleasm download lepidoptera
compleasm protein -t 32 -l lepidoptera -L ~/Desktop/genomes/mb_downloads -p predicted_proteins.fasta -o compleasm-proteins
```

# Field Samples

## Basecall Field samples

```
# Rebasecalled with improved sup
~/Desktop/dorado-0.7.1-linux-x64/bin/dorado basecaller sup LeafMiner_1-9_12-11-23/ -r --kit-name SQK-NBD114-96 --modified-bases 5mCG_5hmCG > LeafMiner_1-9_12-11-23.d0.7.1.multiplex.mod.bam

# Demux the rebasecalled bam
~/Desktop/dorado-0.7.1-linux-x64/bin/dorado demux --output-dir demux --kit-name SQK-NBD114-96 LeafMiner_10-18_12-11-23.d0.7.1.multiplex.mod.bam
```

### Map to reference

```
# Align fastq files made with each field STL collected by host plant and geographic location
# Align to final assembly created above 
mkdir mapped_STL-final
for i in *.bam ; do ~/Desktop/dorado-0.8.0-linux-x64/bin/dorado aligner ../STL-final.fa $i | samtools sort -o $i.mapped.bam -T tmp.ali -@ 32 ; done

# Map Isoline reads to reference
~/Desktop/dorado-0.8.1-linux-x64/bin/dorado aligner STL-final.fa.gz STL_F2_isoline_pooled.5k.herro.fa.gz | samtools sort -o STL_F2_isoline_pooled.5k.herro.mapped.bam -T tmp.ali -@ 32
```

## Variant Analysis

### Clair3 (SNV)

Find variants

```
# Get rerio models
git clone https://github.com/nanoporetech/rerio
./rerio/download_model.py --clair3

# Find some SNVs with Clair3
mamba create -n clair3 -c bioconda clair3 python=3.9.0 -y
mamba activate clair3

# Ran on dorado 0.7.1 sup calls
for i in mapped_STL-final/*.mapped.bam ; do run_clair3.sh --bam_fn=mapped_STL-final/$i --ref_fn=STL-final.fa --threads=32 --platform="ont" --model_path=rerio/clair3_models/r1041_e82_400bps_sup_v430 --output=clair3-out/clair3-$i --sample_name=$i --enable_phasing --include_all_ctgs --fast_mode; done

# Repeat for Isoline F2 modmapped bam

# Merge vcfs and filter for PASS
bcftools merge $(find . -type f -name "merge_output.vcf.gz") --threads 32 --apply-filters PASS -o stl.total.vcf.gz

bcftools merge clair3-SQK-NBD114-96_barcode01.bam.mapped.bam/merge_output.vcf.gz clair3-SQK-NBD114-96_barcode02.bam.mapped.bam/merge_output.vcf.gz clair3-SQK-NBD114-96_barcode03.bam.mapped.bam/merge_output.vcf.gz clair3-SQK-NBD114-96_barcode04.bam.mapped.bam/merge_output.vcf.gz clair3-SQK-NBD114-96_barcode05.bam.mapped.bam/merge_output.vcf.gz clair3-SQK-NBD114-96_barcode06.bam.mapped.bam/merge_output.vcf.gz clair3-SQK-NBD114-96_barcode07.bam.mapped.bam/merge_output.vcf.gz clair3-SQK-NBD114-96_barcode08.bam.mapped.bam/merge_output.vcf.gz clair3-SQK-NBD114-96_barcode09.bam.mapped.bam/merge_output.vcf.gz clair3-SQK-NBD114-96_barcode10.bam.mapped.bam/merge_output.vcf.gz clair3-SQK-NBD114-96_barcode11.bam.mapped.bam/merge_output.vcf.gz clair3-SQK-NBD114-96_barcode12.bam.mapped.bam/merge_output.vcf.gz clair3-SQK-NBD114-96_barcode13.bam.mapped.bam/merge_output.vcf.gz clair3-SQK-NBD114-96_barcode14.bam.mapped.bam/merge_output.vcf.gz clair3-SQK-NBD114-96_barcode15.bam.mapped.bam/merge_output.vcf.gz clair3-SQK-NBD114-96_barcode16.bam.mapped.bam/merge_output.vcf.gz clair3-SQK-NBD114-96_barcode17.bam.mapped.bam/merge_output.vcf.gz clair3-SQK-NBD114-96_barcode18.bam.mapped.bam/merge_output.vcf.gz --threads 32 --apply-filters PASS -o stl.total2.vcf.gz
```

### VCF Filtering

```
#https://www.ncbi.nlm.nih.gov/pmc/articles/PMC10340048/
#For the population genetics analysis, individuals with a high proportion of missing genotypes (missing > 20%, n = 17) were removed. The remaining individuals were used to analyze population differentiation and calculate standard population parameters (heterozygosity, inbreeding coefficient [FIS], effective population size [Ne]); a principal components analysis was employed for visualization purposes. Most calculations were performed using PLINK software [20], though NeEstimator v2.1 was used to estimate effective population size [21], and STRUCTURE software [22] was used to analyze population structure. STRUCTURE was run 10 times per subpopulation (K, from K1 to K3), with 100,000 iterations and a 100,000 burn-in period each. The best K was inferred using StructureSelector software [23] using the method proposed by Evanno et al. [24]. Finally, CLUMPK software was used to visualize the results [25].

# Filtering https://speciationgenomics.github.io/filtering_vcfs/
mamba create --name vcflib_new -c bioconda -c conda-forge vcflib=1.0.3 tabixpp=1.1.0
mamba install -c bioconda vcflib
mamba activate vcflib_new

# Count variants (there are 47,391,733)
bcftools view -H stl.total.vcf.gz | wc -l
bcftools stats stl.total.vcf.gz

# Filter for Q20 according to the speciationgenomics website
# After filtering, kept 13,564,578 (11,186,641 SNPs and 2,546,954 indels)
vcftools --gzvcf stl.total2.vcf.gz --minQ 20 --stdout --recode | bgzip > stl.total2.q20.vcf.gz

# Filter for MAF above 8%, missing below 8%, mean minimum depth of 2X and max depth of 100X.
# MAF cutoff: 18 individuals have 36 alleles, so 8% cutoff means at least 3 individuals must have an allele (36 * n = 3; n=8%) 
# Combined filters, kept 398,561 SNPs. 
vcftools --gzvcf stl.total2.vcf.gz --minQ 20 --remove-indels --maf 0.08 --max-missing 0.9 --min-meanDP 2 --max-meanDP 100 --recode --stdout | bgzip > stl.total2.q20.filt.vcf.gz

# Depth of each individual (ranges from 5X to 25X)
vcftools --gzvcf stl.total2.iso.q20.filt.vcf.gz --depth --out stl.total2.iso.q20.filt.vcf

# Calculate heterozygosity and inbreeding coefficient per individual.
#Computing heterozygosity and the inbreeding coefficient (F) for each individual can quickly highlight outlier individuals that are e.g. inbred (strongly negative F), suffer from high sequencing error problems or contamination with DNA from another individual leading to inflated heterozygosity (high F), or PCR duplicates or low read depth leading to allelic dropout and thus underestimated heterozygosity (stongly negative F). 
vcftools --gzvcf stl.total2.iso.q20.filt.vcf.gz --het --out stl.total2.iso.q20.filt.vcf.gz

# --gvcf - input path – denotes a gzipped vcf file
# --remove-indels - remove all indels (SNPs only)
# --maf - set minor allele frequency - 10% cutoff here
# --max-missing - set minimum non-missing data. A little counterintuitive - 0 is totally missing, 1 is none missing. Here 0.9 means we will tolerate 10% missing data.
# --minQ - this is just the minimum quality score required for a site to pass our filtering threshold. Here we set it to 20.
# --min-meanDP - the minimum mean depth for a site.
# --max-meanDP - the maximum mean depth for a site.
# --minDP - the minimum depth allowed for a genotype - any individual failing this threshold is marked as having a missing genotype.
# --maxDP - the maximum depth allowed for a genotype - any individual failing this threshold is marked as having a missing genotype.
# --recode - recode the output - necessary to output a vcf
# --stdout - pipe the vcf out to the stdout (easier for file handling)
```

## Plink

```
# Linkage pruning
./plink --vcf ../clair3-out/stl.total2.q20.filt.vcf.gz --double-id --allow-extra-chr --set-missing-var-ids @:# --indep-pairwise 50 10 0.1 --out stl.total2.q20.filt

# prune and create pca
./plink --vcf ../clair3-out/stl.total2.q20.filt.vcf.gz --double-id --allow-extra-chr --set-missing-var-ids @:# --extract stl.total2.q20.filt.prune.in --make-bed --pca --out stl.total2.q20.filt

# Plink heterozygosity
# Convert to plink binary "bed" format
./plink --vcf stl.total2.q20.filt.vcf.gz --make-bed --out stl.total2.q20.filt.vcf.gz --allow-extra-chr

# Fst
./plink --bfile stl.q20.filt --fst --out stl.q20.filt.allele_fst --allow-extra-chr

# Allele frequencies
./plink --bfile stl.q20.filt --freq --out stl.q20.filt.allele_freq --allow-extra-chr

# Hardy-Weinberg Equilibrium
./plink --bfile stl.q20.filt --hardy --out stl.q20.filt.hw --allow-extra-chr

# Individual Heterozygosity
./plink --bfile stl.q20.filt --het --out stl.q20.filt.het --allow-extra-chr
```

### Plink PCA

```
# load tidyverse package
library(tidyverse)

# read in data
pca <- read_table2("plink-out/stl.total2.q20.filt.eigenvec", col_names = FALSE)
eigenval <- scan("./plink-out/stl.total2.q20.filt.eigenval")

# sort out the pca data
# remove nuisance column
pca <- pca[,-1]

# set names
names(pca)[1] <- "ind"
names(pca)[2:ncol(pca)] <- paste0("PC", 1:(ncol(pca)-1))

location <- c("Como", "Como", "Como", "Como", "Como", "Como", 
                "Rochester", "Rochester", "Rochester", "Rochester", "Rochester", "Rochester", 
                "Brooten", "Brooten", "Brooten", "Brooten", "Brooten", "Brooten")

species <- c("Hogpeanut", "Hogpeanut", "Hogpeanut", "Soybean", "Soybean", "Soybean", "Hogpeanut", "Hogpeanut", "Hogpeanut", 
             "Soybean", "Soybean", "Soybean", "Hogpeanut", "Hogpeanut", "Hogpeanut", "Soybean", "Soybean", "Soybean")

# Add columns
pca$spp <- species
pca$loc <- location

# combine - if you want to plot each in different colours
spp_loc <- paste0(pca$spp, "_", pca$loc)

# remake data.frame
pca <- as.tibble(data.frame(pca, spp_loc))

# first convert to percentage variance explained
pve <- data.frame(PC = 1:18, pve = eigenval/sum(eigenval)*100)

a <- ggplot(pve, aes(PC, pve)) + geom_bar(stat = "identity")
a + ylab("Percentage variance explained") + theme_light()

# calculate the cumulative sum of the percentage variance explained
cumsum(pve$pve)

# plot pca
b <- ggplot(pca, aes(PC1, PC2, col = spp, shape = loc)) + geom_point(size = 3)
#b <- ggplot(pca, aes(PC1, PC2, col = spp)) + geom_point(size = 3)
b <- b + scale_colour_manual(values = c("red", "blue"))
b <- b + coord_equal() + theme_light()
b + xlab(paste0("PC1 (", signif(pve$pve[1], 3), "%)")) + ylab(paste0("PC2 (", signif(pve$pve[2], 3), "%)"))
```

# Admixture

```
# https://github.com/stevemussmann/admixturePipeline
sudo ./runDocker.sh
admixturePipeline.py -m popmap.tsv -v stl.total2.iso.q20.filt.vcf -k 1 -K 10 -n 32 -t 100 -a 0.05
submitClumpak.py -p stl.total2.iso.q20.filt -M
```

# Phylogeny

```
# Convert to phylip format with vcf2phylip.py
vcf2phylip.py -i stl.total.q20.filt.vcf

# Make tree with iqtree
iqtree -s stl.total.q20.filt.min4.phy -nt 32 -B 1000
```

# MitoHiFi

```
# Pull docker container
sudo docker pull ghcr.io/marcelauliano/mitohifi:master

# Run through singularity
singularity shell --bind /home/cfaulk/Desktop/stl_processing/MitoHiFi:/MitoHiFi docker://ghcr.io/marcelauliano/mitohifi:master mitohifi.py -h
cd MitoHiFi
Singularity> findMitoReference.py --species "Adhemarius gannascus" --outfolder . --min_length 14000
Singularity> mitohifi.py -r ../SQK-NBD114-96_barcode03.bam.mapped.bam.fastq -f ../NC_046728.1.fasta -g ../NC_046728.1.gb -t 32 -o 5

# The "potential contigs" directory lists the contig containing mtDNA (contig_12841).
# Annotations were saved for submission to NCBI Assembly.
```

## Mitotree

Make phylogeny based on mitogenomes of all samples

```
cat *.fasta > mitotree.fasta

# Align with MAFFT
mafft --auto mitotree.fasta > mitotree.aln

# Make tree with iqtree
iqtree2 -s mitotree.aln -nt 32 -B 1000
```

# Methylation

```
# Modkit
~/Desktop/modkit_0.3.1/modkit pileup --ref ../STL_hifiasm-herro-p3-final.fa --cpg SQK-NBD114-96_barcode01.mapped.bam SQK-NBD114-96_barcode01.bed

# Modkit looped
for i in *.bam ; do ~/Desktop/modkit_0.3.1/modkit pileup --ref ../STL_hifiasm-herro-p3-final.fa --cpg $i $i.bed ; done

# Summarize
for i in *.bed; do  awk -v file="$i" '$4=="m" {can+=$13; mod+=$12; oth+=$14; valid+=$10} END{print file "\tCpG canonical " (can/valid) "\tCpG methyl " (mod/valid) "\tCpG hydroxy " (oth/valid)}' $i >> summary.txt; done
```
